# Laminin Receptor 37/67LR Regulates Adhesion and Proliferation of Normal Human Intestinal Epithelial Cells

**DOI:** 10.1371/journal.pone.0074337

**Published:** 2013-08-22

**Authors:** Taoufik Khalfaoui, Jean-François Groulx, Georges Sabra, Amel GuezGuez, Nuria Basora, Patrick Vermette, Jean-François Beaulieu

**Affiliations:** 1 Laboratory of Intestinal Physiopathology, Department of Anatomy and Cell Biology, Faculty of Medicine and Health Sciences, Université de Sherbrooke, Sherbrooke, Quebec, Canada; 2 Laboratory of Bioengineering and Biophysics, Department of Chemical and Biotechnological Engineering, Université de Sherbrooke, Sherbrooke, Quebec, Canada; University Claude Bernard Lyon 1, France

## Abstract

Interactions between the cell basal membrane domain and the basement membrane are involved in several cell functions including proliferation, migration and differentiation. Intestinal epithelial cells can interact with laminin, a major intestinal basement membrane glycoprotein, via several cell-surface laminin-binding proteins including integrin and non-integrin receptors. The 37/67kDa laminin receptor (37/67LR) is one of these but its role in normal epithelial cells is still unknown. The aim of this study was to characterise the expression pattern and determine the main function of 37/67LR in the normal human small intestinal epithelium. Immunolocalization studies revealed that 37/67LR was predominantly present in the undifferentiated/proliferative region of the human intestinal crypt in both the immature and adult intestine. Using a human intestinal epithelial crypt (HIEC) cell line as experimental model, we determined that 37/67LR was expressed in proliferative cells in both the cytoplasmic and membrane compartments. Small-interfering RNA-mediated reduction of 37/67LR expression led to HIEC cell-cycle reduction and loss of the ability to adhere to laminin-related peptides under conditions not altering ribosomal function. Taken together, these findings indicate that 37/67LR regulates proliferation and adhesion in normal intestinal epithelial cells independently of its known association with ribosomal function.

## Introduction

Laminins are the most abundant glycoproteins of basement membranes (BM) both quantitatively and functionally [1,2]. These αβγ heterotrimeric molecules play a role in several cellular processes namely cell growth, migration and differentiation, which are mediated through several types of cell surface laminin receptors [3–5]. These receptors include integrins such as α6β4 [6,7] and α7β1 [8], dystroglycan [9], lutheran [10] and the 37/67 kDa laminin receptor (37/67LR) [11,12].

While 37/67LR was the first laminin receptor to be identified [13,14], its characterization is still incomplete and has been complicated by the fact that it is also involved in a variety of other unrelated roles. Indeed, beside its ability to interact directly with laminin through most likely the CDPGYIGSR sequence on the laminin β1 chain short arm, 37/67LR can play additional roles in the cell. Indeed, phylogenetic analysis carried out on 37/67LR found homologues in all kingdoms from archaebacteria to mammals and suggests that it was originally a ribosomal protein that acquired additional novel functions though evolution [11,15]. As reviewed in detail by Nelson et al. [11], the human 37/67LR gene product (UniGene ID Hs.181357; ribosomal protein name RPSA) has been found in the ribosome of all tissues investigated [16] where it appears to serve as a critical component of the translational machinery [17]. The 37/67 kDa laminin receptor has also been identified as a component of the nuclear machinery where it can bind to both chromatin and the nuclear envelope [18–20]. It is noteworthy that 37/67LR can also act as a cell surface receptor for bacteria, viruses and prions [11,12,21]. Consistent with these multiple functions, 37/67LR is not only localized on the cell surface but can also be found in the cytoplasm, perinuclear compartment and nucleus. The alternative "37/67 kDa" nomenclature still used to identify 37/67LR arises from the observation that the gene corresponding to the originally identified 67 kDa laminin-binding protein encodes a 32.8 kDa protein, which migrates at 37 kDa on SDS-PAGE suggesting that the 67 kDa form could result from homo or heterodimerization reactions involving the 37 kDa precursor and fatty acid acylation [22–24]. Albeit the proposed precursor-product relationship, the exact relationship between the 37LR precursor and 67LR remains unclear [11,12,21]. For instance, some antibodies raised against amino-peptides of the 37 kDa sequence failed to recognize the 67 kDa polypeptide in Western blots [25] while the 37LR precursor can be detected on the plasma membrane [25–27].

Functionally, 37/67LR has attracted considerable interest since its discovery 30 years ago [28,29]. Indeed, over-expression of 37/67LR has been shown in a variety of cancer cell types where its expression levels have been found to strongly correlate with the risk of tumour invasion and metastasis [30–33]. 37/67LR may also be of importance in other pathologies including neurodegenerative and angiogenic diseases such as Alzheimer’s disease [21] and retinal neovascularisation [34]. The mechanism has not yet been elucidated but recent studies indicate that 37/67LR can prevent apoptosis [35,36] and acts as the cell receptor that mediates the anti-inflammatory and anti-thrombotic activities of epigallocatechin-3-gallate [37–39]. Further studies are nevertheless required to fully understand the involvement of 37/67LR in these pathologies [11].

Another intriguing question pertaining to 37/67LR is its role in the normal state. Indeed, very few studies have addressed its extraribosomal function in normal cells [11]. The intestinal epithelium represents a useful system to investigate such a question. Indeed, under physiological conditions, the architecture of the small intestinal mucosa is maintained through a sensitive equilibrium between epithelial cell production and maturation in the crypt compartment and migration along the length of the crypt-villus axis and extrusion at the villus tips [40–42]. As epithelial cells migrate upwards they are exposed to compositional changes of the underlying BM components such as collagens, fibronectin and laminins [41,43–45], which have been demonstrated to influence cell behaviours including proliferation, migration, differentiation and cell survival [46–52]. Interestingly, 37/67LR expression has previously been investigated along the intestinal epithelial crypt-villus axis. While the analysis performed on the human normal intestinal epithelium revealed relatively contradictory staining patterns from weak/absent [53] to predominant expression in the brush border/supranuclear region of the entire epithelium [54] or in the basolateral domain restricted to crypt epithelial cells [55], transcript analysis in the rat intestine revealed higher mRNA levels of 37/67LR in the fetal intestine and adult epithelial crypt cells suggesting that high 37/67LR expression may correlate with proliferation in intestinal cells [33].

In the present study, we sought to determine the role of 37/67LR on normal cell functions using normal human small intestinal specimens as well as a human intestinal epithelial crypt (HIEC) non-transformed non-immortalized cell line to perform experimental analysis. By immunofluorescence, we first established the predominant expression of 37/67LR in the immature intestinal epithelium and in the crypt cells of the adult small intestine and confirmed that these observations can be extended to proliferating HIEC cells. Then, using an siRNA strategy under conditions that down-regulate 37/67LR expression without altering translation, we found a significant reduction of both HIEC cell proliferation and specific adhesion to the laminin-related peptide YIGSR. Taken together, these data establish for the first time that 37/67LR expression in normal intestinal epithelial cells regulates cell proliferation and adhesion, two crucial functions for intestinal epithelial homeostasis.

## Materials and Methods

### Antibodies and reagents

A rabbit polyclonal anti-human 37/67LR antiserum (ab90073, Abcam, Burlington, ON) was used at a 1:1000 dilution for both indirect immunofluorescence and Western blotting in all experiments. Monoclonal blocking anti-37/67LR MLuC5 azide-free antibody (Abcam) was used at 20 µg/ml in blocking experiments. The mouse monoclonal antibody anti-fibronectin (HFN 7.1, Developmental Studies Hybridoma Bank, Iowa City, IA) was used at a 1:500 dilution for Western blot. The rabbit polyclonal antibody against the Paneth cell specific phospholipase A2 (sc20105, Santa Cruz Biotechnology, Santa Cruz, CA) was used at a 1:5000 dilution in immunofluorescence. Monoclonal antibodies against Ki67 and β-actin were used at 1:500 and 1:80,000, respectively (MAB4190 and MAB1501, Millipore, Burlington, ON) while the anti-integrin β1 antibody Mab13 was used at 1:500 for Western blot and 20 µg/ml in blocking experiments (552828, BD Biosciences, Mississauga, ON). For WB, the rabbit polyclonals anti-H3K27me3 (07-449, Millipore), anti-p27 (sc528, Santa Cruz Biotechnologies) and anti-TIMP3 (AB802, Millipore) were used at 1:1000 dilution and the mouse monoclonals anti-cytokeratin 18 (CY-90, Sigma-Aldrich, Oakville, ON) and anti-IGFBP7 (R&D Systems, Minneapolis, MN) were used at 1:10000 and 1:1000, respectively. Secondary antibodies used were goat anti-mouse AlexaFluor 488, goat anti-rabbit AlexaFluor 594 and goat anti-rat AlexaFluor 568 (Invitrogen, Burlington, ON) for immunofluorescence and horseradish peroxidase-conjugated secondary antibodies (anti-mouse, anti-rabbit, Amersham-GE Healthcare, Baie d’Urfe, QC) for Western blot.

### Tissues

Tissues from the normal adult proximal ileum were obtained from Quebec Transplant. Specimens of small intestine (ileum) were obtained from fetuses ranging in age from 10 to 20 weeks of gestation following legal or therapeutic pregnancy termination with written informed patient consent. No tissues were collected from cases associated with known fetal abnormalities or intrauterine fetal demise. Studies were approved by the Institutional Review Committee for the Use of Human Material of the “Centre Hospitalier Universitaire de Sherbrooke/Faculté de Médecine et des Sciences de la Santé”. In some experiments, pure epithelial and stromal fractions were prepared and analysed as previously described [56].

### Indirect immunofluorescence staining

The preparation and embedding of tissue samples for cryosectioning were performed as described previously [57]. Cryosections (3-µm thick) were prepared, fixed, and stained for indirect IF as previously described [46]. Both primary and secondary antibodies were diluted in PBS (pH 7.4) containing 5% BLOTTO or 2% BSA. Nuclei were counterstained 2 min at room temperature with 10 ng/ml 4’6-diamidino-2-phenylindol dihydrochloride (DAPI)-PBS (pH 7.4). Samples were mounted in glycerol–PBS (9:1) containing 0.1% phenylenediamine and viewed with a DMRXA microscope (Leica) equipped for epifluorescence and digital imaging (RTE/CCD Y/Hz-1300 cooled camera).

### HIEC cell culture

Human intestinal epithelial crypt HIEC cells are normal non-transformed non-immortalized cells that have been characterized elsewhere [58,59] and extensively used as a human intestinal crypt cell model [60]. Briefly, they express typical keratins and specific crypt cell markers (keratins 18 and 20, dipeptidylpeptidase IV, the crypt cell antigen MIM-1/39, etc.) confirming their intestinal epithelial origin [58,59,61]. Typical cell markers identified in the lower crypt in the intact intestine are also expressed in HIEC cells including adhesion and signaling molecules [61–63], metabolism [64] and inflammatory response [65,66]. Furthermore, while HIEC cells are undifferentiated, studies have shown that reintroduction of the intestine-specific pro-differentiation factors CDX2, HFN-1α and GATA-4 can induce enterocytic differentiation [67,68]. Taken together, HIEC cells represent a valid human intestinal crypt cell model.

### RNA interference and transfection

Predesigned siRNA sequences targeting 37/67LR (siLR1, 2, 3 and 4) and a non-silencing negative control siRNA (siCtrl) were purchased from Sigma Aldrich (Oakville, ON) and used as previously described [46]. One day prior to transfection, 2x10^5^ cells were plated in 30mm dishes (Falcon Plastics) and transfected with siRNAs using the X-tremeGENE siRNA transfection reagent (Roche Diagnostics, Laval, QC). Cells were used in the various experiments 48 hours post-transfection unless otherwise specified.

### RNA extraction and RT-PCR

Total RNA was extracted using the TRIzol Reagent (Life Technologies, Burlington, ON) according to the manufacturer’s instructions. RT-PCR was performed as described previously [56]. Primers used were: for 37/67LR, LR-AS: 5’-gcagcagcctgctcttctttt-3’, and LR-S: 5’-gagctcactcagtgggtttgatg-3’; for tenascin, TNC-F: 5’ accacaatggcagatccttc-3’, and TNC-R 5’- gcctgccttcaagatttctg 3’; for E-cadherin, Ecad-1: 5’-ccttcctcccaatacatctccc, and Ecad-2: 5’-tctccgcctccttcttcatc; for RPLPO (ribosomal protein, large, P0), RPLPO-F: 5’-gcaatgttgccagtgtctg, and RPLPO-R: 5’-gccttgaccttttcagcaa. Each cycle was composed of template denaturation at 94°C for 45 sec, primer annealing at 60°C for 45 sec, and elongation at 72°C for 1 min for 30 cycles. RT-PCR experiments were performed using an iCycler™ Thermal Cycler (BioRad, Mississauga, ON).

### Real-time RT-PCR quantification analyses

For quantitative PCR experiments, primers for 37/67LR and RPLPO were the same as above. Amplification efficiencies and assessment of differences in gene expression between control and experimental conditions were established according to the Pfaffl mathematical model [69]. Relative mRNA expression levels were established by comparing the levels under experimental conditions to those of control samples and RPLPO was used for normalization. For quantitative evaluation of transcript levels, real-time experiments were performed using an Mx3000P (Stratagene, La Jolla, CA) as previously described [70].

### Western blotting analyses

Western blots were performed on SDS-PAGE gels under reducing conditions as previously described [46,49]. Total proteins (50µg/ml) were separated on 12% SDS-PAGE gels, electrotransferred onto a nitrocellulose membrane (BioRad) and probed as described previously [46]. Immunoreactive bands were visualized using the Immobilon Western kit (Millipore) according to the manufacturer’s instructions or with AlexaFluor secondary antibodies and visualized with a Molecular Imager® FX equipped with an external laser (BioRad).

### Epithelial-stromal dissociation

Pure fractions of epithelial and mesenchymal cells from mid-gestation ileum were obtained using Matrisperse (BD Biosciences) and tested as previously described [56,60].

### Cellular fractionation

The Subcellular Proteome Extraction Kit (Millipore) was used to sequentially extract subcellular compartments (cytosolic: F1; membrane: F2; nuclear: F3; cytoskeletal: F4) for Western blot analyses. Enriched fractions were verified by the detection of p27KIP1 (cytoplasmic soluble fraction), β1 integrin subunit (membrane fraction), trimethylated lysine 27 on histone 3 (nuclear fraction) and keratin 18 (cytoskeletal fraction).

### BrdU incorporation assays

BrdU incorporation experiments in cells were performed in accordance with the In Situ Cell Proliferation Kit FLUOS® protocol (Roche) as described previously [71].

### Cell-cycle progression analysis by laser scanning cytometry

HIEC were seeded onto 12-well plates (Falcon) at 5× 10^4^ cells per well 48 h under standard culture conditions with complete medium before methanol fixation and DAPI staining. DAPI-stained cells were scanned with an iCys imaging cytometer (Compucyte, Cambridge, MA) to measure DNA content using violet diode laser excitation (405 nm) and emission (460 nm) filters for fluorescence detection as previously described [68]. DNA content was measured for at least 3000 isolated nuclei per sample in three separate experiments to assess cell-cycle distribution.

### Bioactive surface preparation and cell adhesion assay

Polystyrene 12-well tissue culture plate surfaces were prepared for grafting CDPGYIGSR and GRGDSPC (American Peptide Company, Cat. 87-0-53 and 44-0-25, Sunnyvale, CA) on CMD low-fouling surfaces using maleimide chemistry as previously described [72].

Preparation of CMD, GRGDSPC and CDPGYIGSR surfaces and cells prior to seeding was as described [73]. Briefly, HIEC cells were harvested using PBS/0.5 mM EDTA 48 hours after transfection. Cells were seeded (1×10^5^ cells per well) and incubated for 1 h. Non-adherent cells were gently washed away with PBS and the remaining cells were fixed with 2.0% paraformaldehyde (pH 7.4) for 25 min at 4°C and permeabilized with 0.1% Triton X-100. Cell nuclei were stained with DAPI and adherent cells were counted manually in 10 squares of 0.42 mm^2^.

### Blocking experiments

HIEC were harvested using PBS/0.5 mM EDTA for 10 min and processed as described previously [71] for blocking experiments. Briefly, HIEC cells were resuspended in serum-free culture medium containing 20 µg/ml of neutralizing antibody or non-immune serum protein for 1h, then seeded in complete culture medium onto 12 well plates and incubated at 37^o^C for 24h before being processed for BrdU staining.

### Statistical analysis

Results are expressed as mean ± S.E.M. Each experiment was repeated at least three times and representative results are shown. Student’s paired t-test and ANOVA using Bonferroni’s Multiple Comparison Test were used to analyze the data. Data were considered to be statistically significant at p < 0.05. Statistical calculations were performed using Prism 3.0 Software (GraphPad Software, San Diego, CA).

## Results

### In situ distribution of 37/67LR in the human small intestinal mucosa

**Figure 1:**
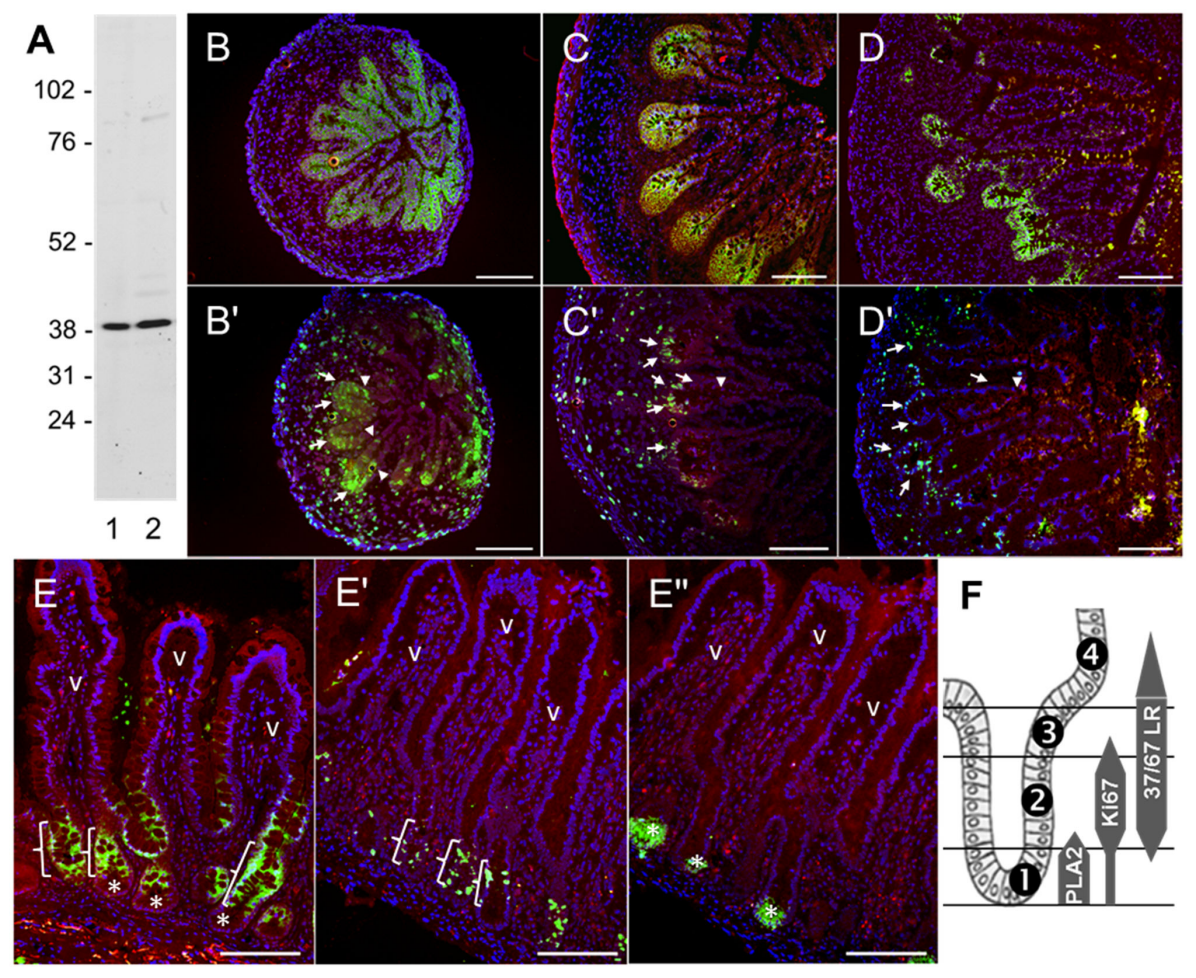
Expression of 37/67LR in normal human intestinal cells. As shown by Western blot (A), the anti-37/67LR antibody predominantly detects a major band at ~37 kDa in normal HIEC cells (lane 1) as well as in the positive control Caco-2 cells (lane 2). In the developing human small intestine (B–D) immunolocalization of 37/67LR revealed predominant expression of the receptor in the intervillous area at 10 weeks of gestation (B) and in the crypts at 14 (C) and 20 weeks (D) coinciding with the corresponding proliferative compartment as defined by the proliferative antigen Ki67 on serial sections (arrows in B', C' and D'). In the adult, 37/67LR (E) was mainly located in the middle part of the crypts (brackets) containing the most actively proliferating cells as revealed by Ki67 (E'). Villus (v) cells as well as the Paneth/stem cell region (*), the latter defined by the expression of the Paneth cell marker PLA2 (* in E'') were both negative for 37/67LR. Immunostaining of the stromal compartment was below the detection level at all stages. Evan blue was used as a histological counterstain (brown/red). Nuclei were stained with DAPI (blue). Scale bars in A-D: 50 µm; E: 100 µm. (F) Summary of observations in the adult small intestine where expression of 37/67LR was consistently found to be absent from the Paneth/stem cell zone (1), maximal in the middle crypt region containing the Ki67-positive transit amplifying cells (2) and decreased toward the terminal differentiation compartment (3) and the base of the villus (4).

The expression and distribution patterns of 37/67LR along the crypt-villus axis in the normal human fetal and adult small intestinal mucosa were determined by indirect immunofluorescence on cryosections. The specificity of the antibody was determined by Western blot. As shown in Figure 1A, the anti-37/67LR antibody predominantly detected a major band at the expected molecular range of the monomer, i.e. ~37 kDa, in normal HIEC cells (Figure 1A, lane 1) as well as in Caco-2 cells (lane 2), a colorectal cancer cell lines used here as positive control. No specific band was detected in the 67 kDa range with this antiserum (Figure 1A) or with three other tested anti-37/67kDA antisera (not shown). At 10 weeks of gestation, crypts are not yet formed [41] and 37/67LR staining was found to be widespread in both the intervillous region and the villus (although at a weaker intensity) (Figure 1B). As expected at this stage, cell proliferation as evaluated by Ki67 staining was found in the intervillous area and in the lower part of the villus (Figure 1B', arrows and arrowheads, respectively). At 14 and 20 weeks (Figure 1C and D), 37/67LR staining was gradually restricted to the crypts where the proliferative epithelial cells are confined (Figure 1C' and D', arrows). In the adult, expression of 37/67LR was also predominantly located to the middle and upper parts of the crypts (Figure 1E, brackets) that also contains the most active proliferative cell population (transit amplifying cells) as identified with Ki67 (Figure 1E'). The villus differentiated epithelium (v) as well as the lower part of the crypts (*) containing the differentiated PLA2-expressing Paneth cells (Figure 1E'') were found to be negative (Figure 1E). As summarized in Figure 1F based on a series of observation, the 37/67LR was consistently found to be absent from the Paneth/stem cell zone (1). Its expression was maximal in the middle crypt containing the Ki67-positive transit amplifying cells (2) while its expression decreased toward the terminal differentiation compartment (3) and the base of the villus (4).

**Figure 2:**
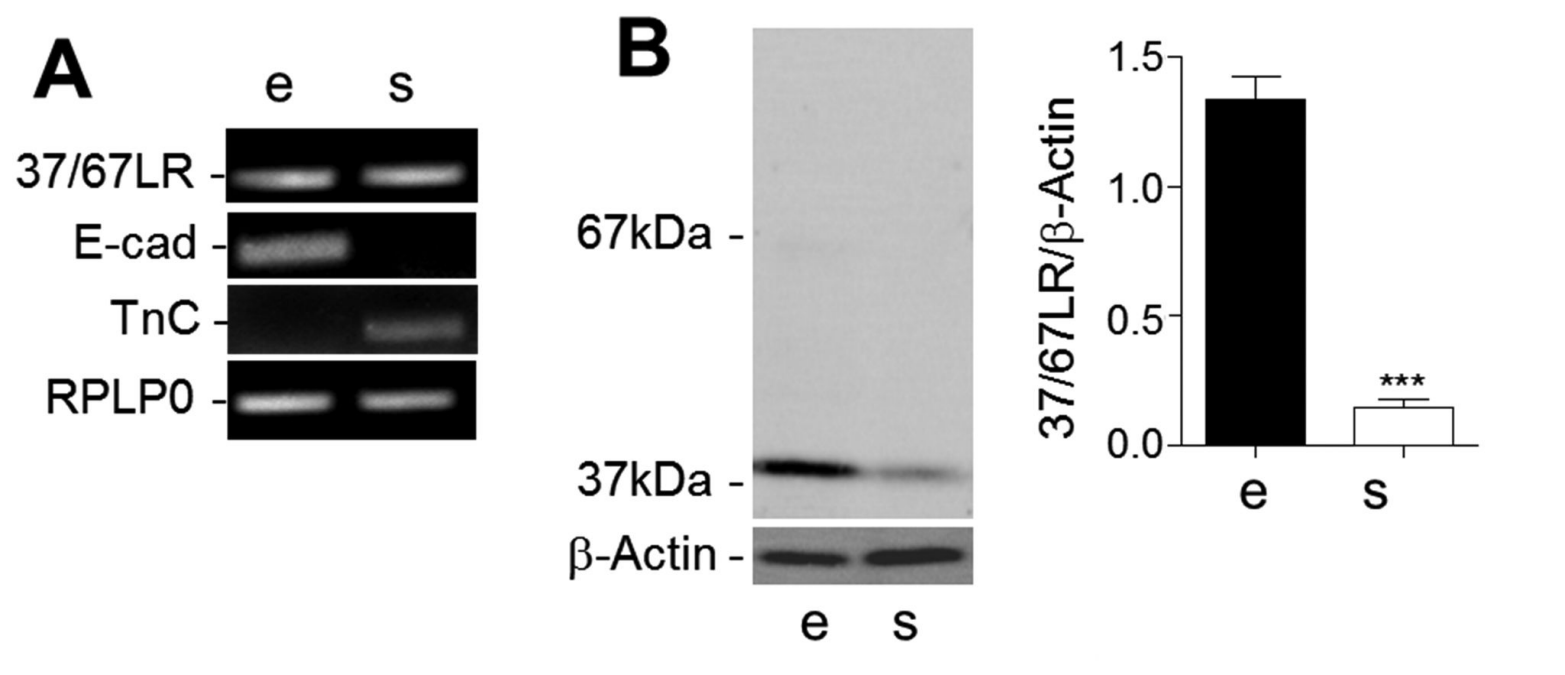
Tissular expression of 37/67LR in the intestine. (A) Representative semi-quantitative PCR analysis of purified epithelial (e) and stromal (s) fractions showed that the 37/67LR transcript can be found in both tissues. RPLPO served as the normalizer. The purity of each fraction was shown by the detection E-cadherin (E-cad: epithelial) and tenascin-C (TnC: stromal). (B) Western blot analyses confirmed the presence of 37/67LR in both the epithelial and stromal fractions by detecting the 37kD component although at higher amounts in the epithelial fraction relative to β-actin (mean ± SEM, n=3, ***: p < 0.0005).

To confirm this distribution, the expression of 37/67LR was analyzed on purified epithelial and stromal fractions isolated from mid-gestation ileum based on a method established in our laboratory [56]. Semi-quantitative RT-PCR showed the presence of the 37/67LR transcript in both fractions (Figure 2A). The purity of the fractions was confirmed by the exclusive expression of E-cadherin (epithelial) and tenascin-C (stromal). Analysis of 37/67LR at the protein level confirmed the presence of 37/67LR in its 37 kDa form in both fractions (Figure 2B) although quantitative assessment showed ~6 times more antigen in the epithelium than in the stroma (Figure 2C). Taken together, these observations indicate that 37/67LR is predominantly expressed in mucosal epithelial cells and proliferative cells in both the fetal and adult intestine.

### Proliferative Human Intestinal Epithelial Crypt Cells Express 37/67LR In Vitro

**Figure 3:**
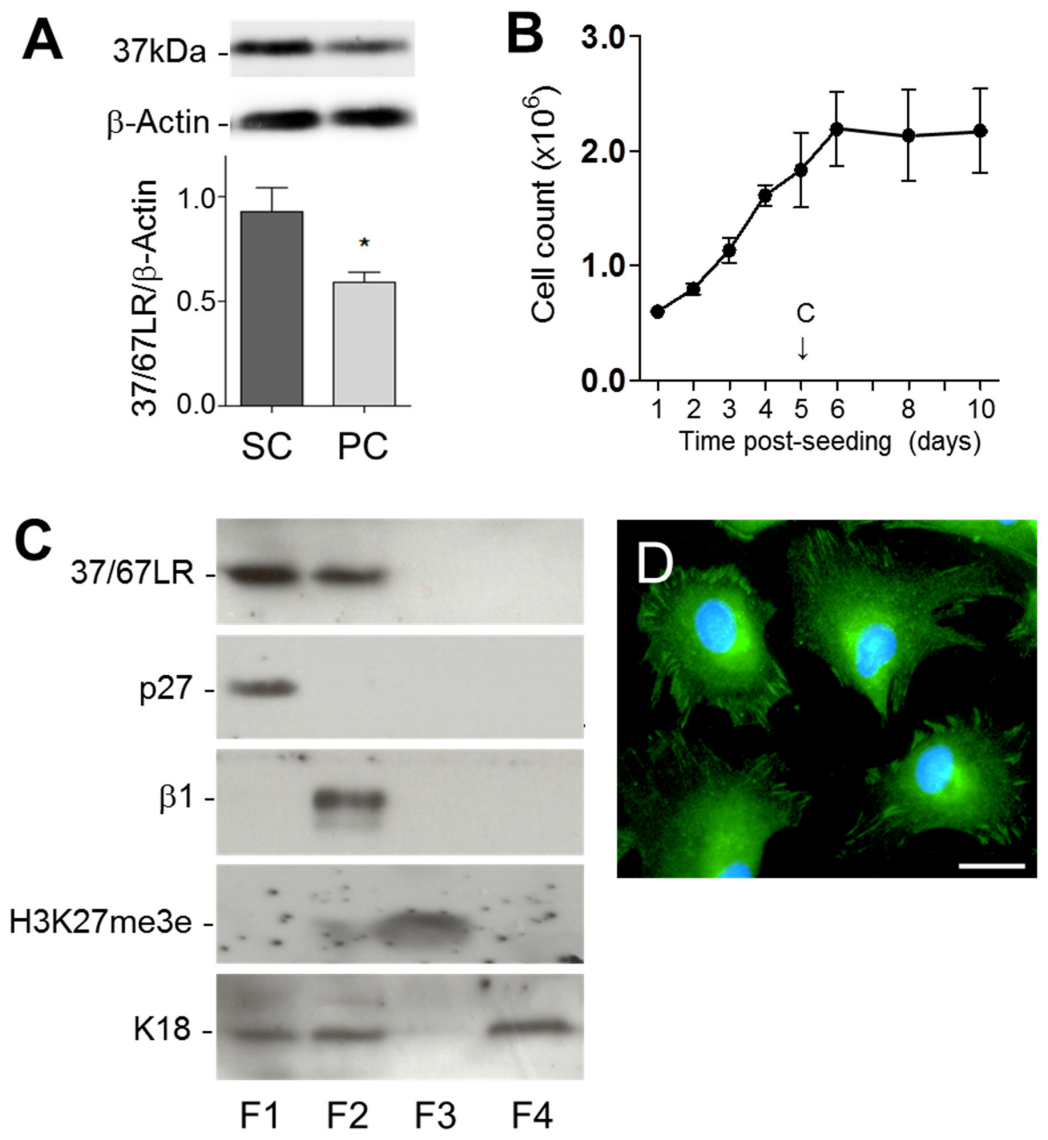
Characterization of 37/67LR in human intestinal epithelial crypt (HIEC) cells. (A) Western blot analysis of subconfluent (SC) and 5 day postconfluent (PC) HIEC cells revealed expression of 37/67LR in proliferative cells and a significant reduction of its expression in quiescent cells relative to β-actin (mean ± SEM, n=3, *: p < 0.02). (B) Cell counts over a 10 day period after seeding confirmed that cell number stabilizes after confluence (c ->). (C) Representative Western blot analysis of 37/67LR in enriched HIEC cytoplasmic (F1), membrane (F2), nuclear (F3) and cytoskeletal (F4) fractions showing that 37/67LR was essentially found in the cytoplasmic and membrane fractions. Enriched fractions were verified by the detection of p27^KIP1^ (p27; cytoplasmic fraction F1), the β1 integrin subunit (β1; membrane fraction F2), trimethylated lysine 27 on histone 3 (H3K27me3e; nuclear fraction F3) and keratin 18 (K18; cytoskeletal fraction F4; also detectable in the cytoplasmic F1 and membrane F2 fractions). (D) Indirect immunofluorescence for the detection of 37/67LR in HIEC cells showing mainly perinuclear granular and weak membrane staining. Nuclei were stained with DAPI (blue). Scale bar in D: 50 µm.

Considering the distribution pattern of immunoreactive 37/67LR in intestinal crypts in situ, we used the normal human intestinal crypt (HIEC) cell line to further investigate 37/67LR expression in relation to cell proliferation. As shown in Figure 3, 37/67LR expression was observed in actively proliferating HIEC cells (Figure 3A, SC) and a significant reduction was observed in HIEC cells maintained for 5 days at confluence (Figure 3A, PC). Indeed, while subconfluent HIEC cell growth is linear before confluence, proliferation is totally inhibited after confluence (Figure 3B).

Cell fractionation of HIEC cells was then performed in order to further characterize 37/67LR expression in HIEC cells. As shown in Figure 3C, immunoreactive 37/67LR was predominantly found in the enriched cytosolic (F1) and membrane (F2) fractions while being below detection levels in the nuclear (F3) and cytoskeletal (F4) fractions. Detection of p27^KIP1^ (p27), the β1 integrin subunit (β1), trimethylated lysine 27 on histone 3 (H3K27me3e) and keratin 18 (K18) were used to confirm fractionation. Immunodetection of 37/67LR by indirect immunofluorescence confirmed cytoplasmic and membrane-like associated staining (Figure 3D).

### Knockdown of 37/67LR expression under conditions that do not affect protein translation

**Figure 4:**
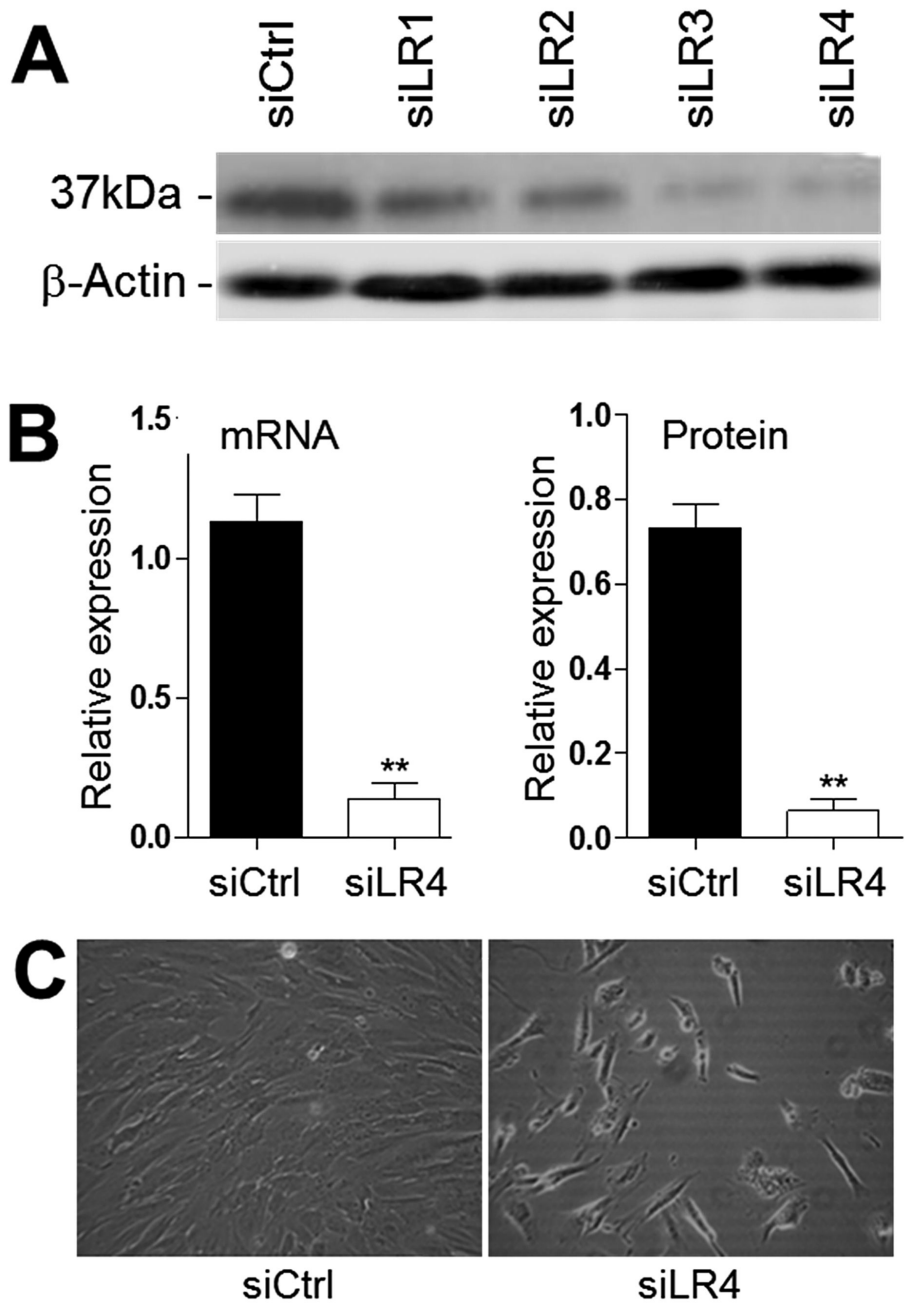
Knockdown of 37/67LR expression in HIEC cells. (A) Representative Western blot analysis showing the expression level of 37/67LR following a period of 48h after transfection of the 4 predesigned siRNA sequences (siLR1-4) and control si (siCtrl). All sequences induced a significant reduction of 37/67LR, but siLR4 was most efficient. (B) Representative graphs showing relative amounts of 37/67LR mRNA by qPCR and protein by Western blot in control (siCtrl) and knockdown (siLR4) HIEC cells. Relative amounts were obtained from data normalized with RPLPO (mRNA) and β-actin (protein) (mean ± SEM, n=3, **: p<0.0005). (C) Phase contrast micrographs of HIEC cells taken 48h after transfection with siCtrl and siLR4.

As a first step to investigate the function of 37/67LR in HIEC cells, we transiently knocked down its expression by transfection with siRNA targeting the 37/67LR sequence. Analysis of 37/67LR expression after 48 hours in the presence of 40 µM of one of 4 predesigned siRNA sequences (siLR1,2,3 and 4) and siCtrl was performed by Western blot (Fig. 4A). While all 4 sequences tested showed some inhibition of expression relative to control, siLR4 showed more efficiency and reproducibility. Using siLR4, 37/67LR expression was found to be reduced by more than 5 times at both the transcript and protein levels (Figure 4B) while the appearance and viability of the cells appeared to be altered under these conditions (Figure 4C).

**Figure 5:**
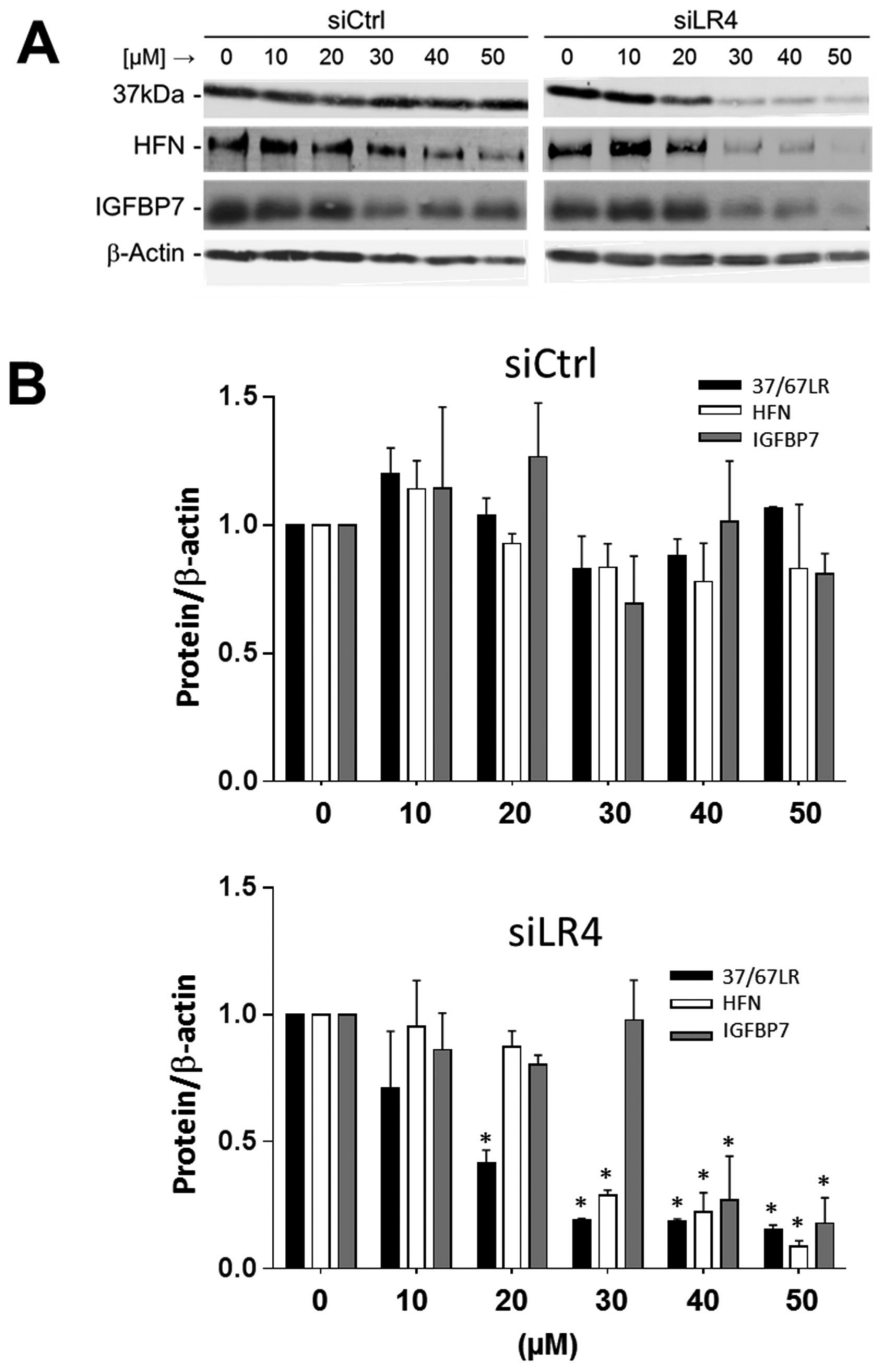
Reduction of 37/67LR expression without affecting endogenous protein translation. (A) Representative western blot analysis of 37/67LR (37kDa), human fibronectin (HFN), insulin like growth factor binding protein 7 (IGFBP7) and β-actin in HIEC cells 48h after transfection with siCtrl and siLR4 at various concentrations (0-50 µM). (B) Relative 37/67LR, HFN and IGFPB7 expression were found to be stable in the presence of increasing concentrations of siCtrl. With siLR4, 50% knockdown of 37/67LR expression was achieved at 20 µM, a concentration that had no effect on HFN or IGFBP7 expression in contrast to higher concentrations such as 40 and 50 µM which resulted in more than 80% knockdown of 37/67LR expression and inhibition of protein synthesis as observed for HFN and IGFBP7. Relative amounts of 37/67LR, HFN and IGFBP7 were determined by optical densitometry and expressed relative to β-actin (mean ± SEM, n=3 separate experiments, * p < 0.05 relative to 0 µM)).

Because 37/67LR is thought to be required for protein translation [35], we performed a 0-50 µM dose–response analysis of siLR4 efficiency in order to evaluate 37/67LR expression relative to other control gene products such as fibronectin, IGFBP7 and TIMP3 which are constitutively produced by HIEC cells [47,71,74] and used to monitor translation. Control siRNA (siCtrl) dose–response showed that expression of 37/67LR, fibronectin, IGFBP7 and TIMP3 was not altered at any tested concentration (Figure 5A, left panel; Figure 5B, upper panel; TIMP3 data: 1.0 at 0 µM vs 1.05 ± 0.15 at 20 µM). In contrast, siLR4 showed maximal apparent efficiency at 30 µM and higher for both 37/67LR and fibronectin and 40 and 50 µM for IGFBP7 (Figure 5A, right panel). However at 20 µM, a ~50% knockdown of the expression of 37/67LR was observed with siLR4 while neither fibronectin, IGFBP-7 nor TIMP3 expression was significantly affected (Figure 5B, lower panel; TIMP3: 1.0 at 0 µM vs 1.22 ± 0.14 at 20 µM). These results indicated that siLR4 at a concentration of 20µM (siLR4-20) can significantly reduce 37/67LR expression without altering general translation. We then used these conditions to investigate the role of 37/67LR in normal HIEC cells.

### Reduction of 37/67LR expression inhibits cell proliferation in G1

**Figure 6:**
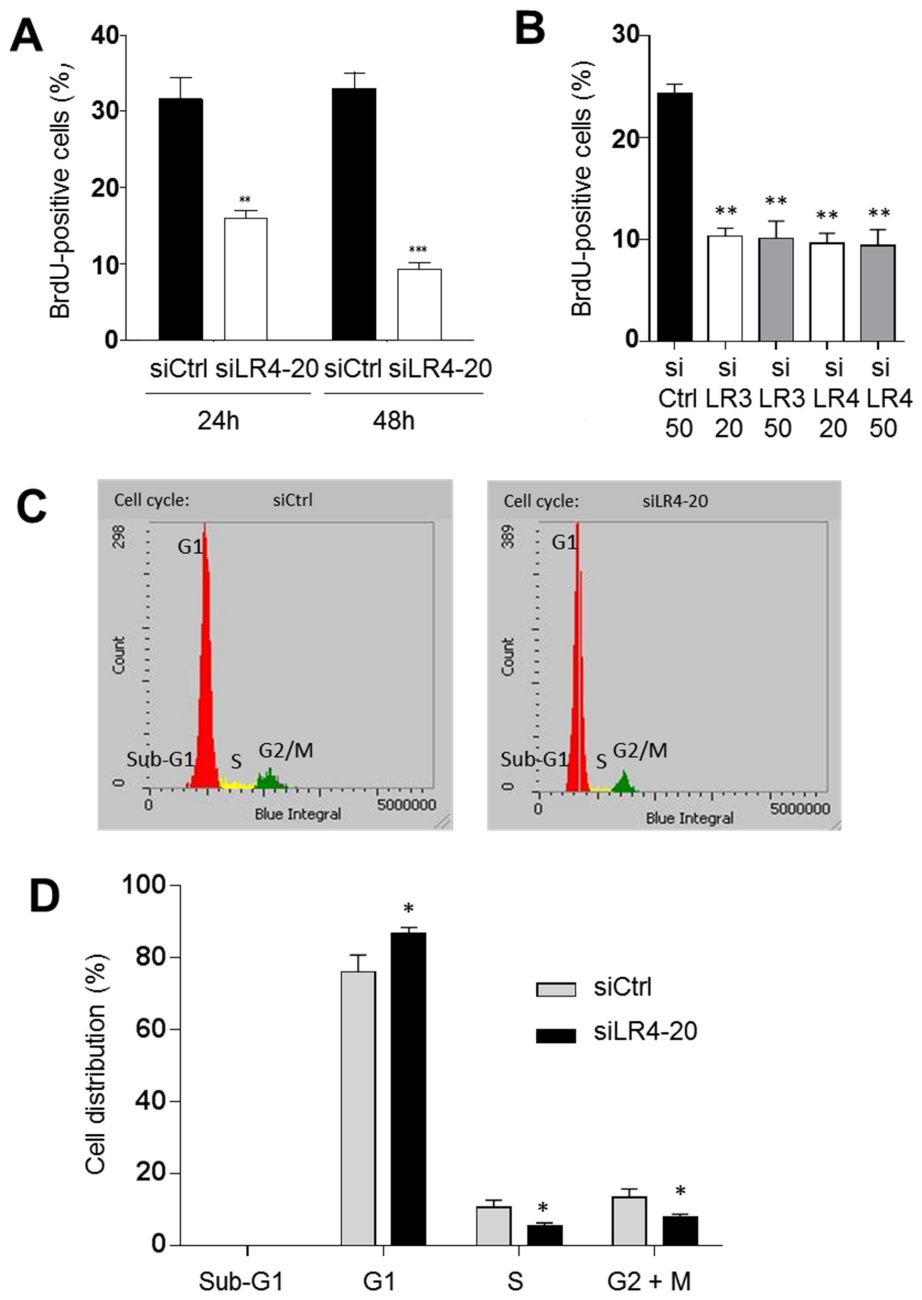
Reduction of 37/67LR expression inhibits cell proliferation. (A) Proliferation was evaluated in HIEC cells 24 and 48h after transfection with siCtrl or siLR4 at 20 µM (siLR4-20) by BrdU incorporation. Transfected cells were seeded on plastic and stained using an anti-BrdU antibody (mean ± SEM, n=3, ** p< 0.005; *** p < 0.001). (B) The specificity of the inhibitory effect of a reduction of 37/67LR on BrdU incorporation was confirmed on HIEC treated during 48h with siCtrl (50µM), siLR3 and siLR4 (both at 20 and 50 µM) (mean ± SEM, n=3, ** p < 0.001). (C) Representative iCys laser scanning cytometric images from a single experiment exhibiting changes in the progression of cell cycle in HIEC cells 48h after transfection with siCtrl or siLR4 at 20 µM (siLR4-20). G1, S, and G2/M in each micrograph represent the percentage of cells present in normal phases of the cell cycle whereas Sub-G1 represents the percentage of cells that have undergone apoptosis. (D) Histogram illustrating the percentage (%) of cells distributed in each of Sub-G1, G1, S and G2+M cell-cycle phases after 48 h for HIEC control (siCtrl) and knockdown (siLR4-20) obtained by iCys laser scanning cytometry analysis (mean ± SEM, n=3, * p < 0.05).

To determine whether 37/67LR was directly implicated in cell proliferation, we first evaluated proliferation by BrdU incorporation 24 and 48h following control and siLR transfection. As expected from previous studies, BrdU incorporation under these experimental conditions resulted in a ~30% proportion of positive cells under control conditions [46,71] as also observed herein (Figure 6A). A significant reduction of cell proliferation was observed at both 24 and 48h after transfection with the siLR4 used at 20 µM (Figure 6A). To confirm the specificity of the effect, we tested a second siRNA. As shown in Figure 6B, siLR3 used at both 20 and 50 µM had comparable effects on inhibition of BrdU incorporation as siLR4.

To further characterize the involvement of 37/67LR on HIEC proliferation, laser scanning cytometry analysis revealed that the apparent decrease in DNA synthesis observed in Figure 6A was consistent with a significant increase in the percentage of cells in G1 and a ~ 50% reduction in S phase in siLR4-20 treated cells as compared to control (Figure 6C and D) after 48 h. It is noteworthy that the lack of staining in the Sub-G1 region for both siCtrl and siLR4 (Figure 6C and D) indicates that apoptosis was not involved.

### Reduction of 37/67LR expression inhibits LM-111 (YIGSR)-dependent cell adhesion

**Figure 7:**
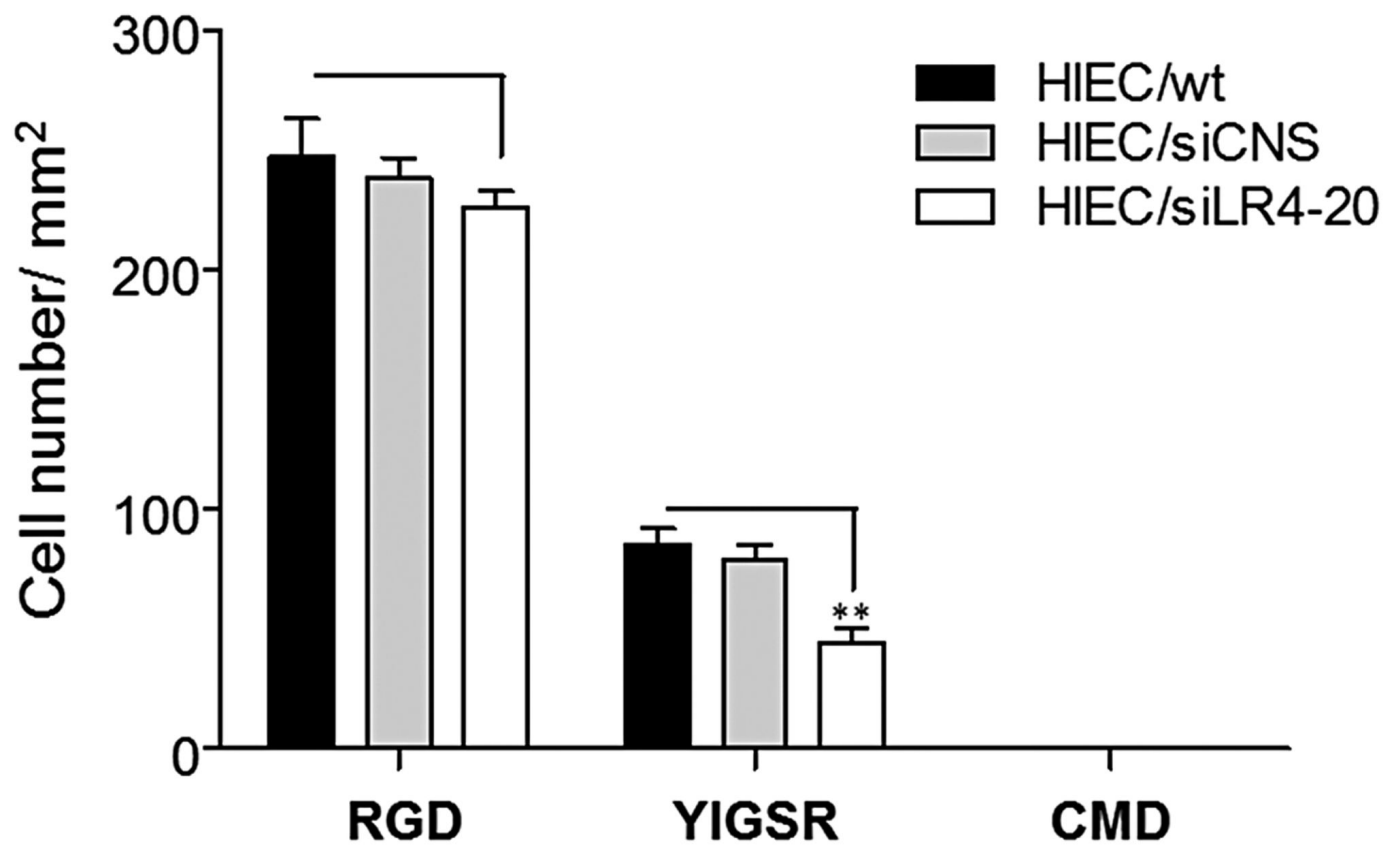
Decreased expression of 37/67LR results in reduction of cell adhesion to LM-111 YIGSR related peptide. Controls and HIEC cells exhibiting reduced 37/67LR expression (siLR4-20) were used for 1h adhesion assays on low-fouling carboxymethyl-dextran (CMD) layers bearing the tripeptide Arg–Gly–Asp (RGD) or CDPGYIGSR, a laminin nonapeptide (YIGSR). The CMD surface prevents cell adhesion. HIEC cell adhesion to RGD was maximal and comparable for control and siLR-treated cells whereas a statistically significant reduction of cell adhesion on YIGSR was observed in partially 37/67LR-depleted cells (mean ± SEM, n=3, ** p < 0.01).

Previous studies have identified that 37/67LR binds to the short arm of the laminin β chain via the YIGSR peptide sequence [11]. This characteristic was used to evaluate whether 37/67LR functions as a laminin-111 receptor in normal intestinal epithelial cells. Cell adhesion assays were performed on low-fouling surfaces on which peptide immobilization supports specific cell-biomaterial interaction while preventing non-specific protein adsorption allowing the discrimination of a specific from a non-specific cell response [72]. As performed previously [73], non-specific adhesion was determined on CMD surfaces which prevent cell adhesion, 37/67LR-mediated adhesion was evaluated on CDPGYIGSR (YIGSR) while maximal adhesion was determined on RGD peptide. Previous studies have shown that HIEC cells attach strongly to RGD peptides [71]. The results showed that reduced 37/67LR expression had no significant effect on binding to the RGD peptide but induced a statistically significant ~50% reduction of adhesion to YIGSR (Figure 7) confirming that 37/67LR is functional for LM-111 adhesion in intestinal crypt cells.

### Functional blocking of the 37/67LR inhibits cell proliferation

**Figure 8:**
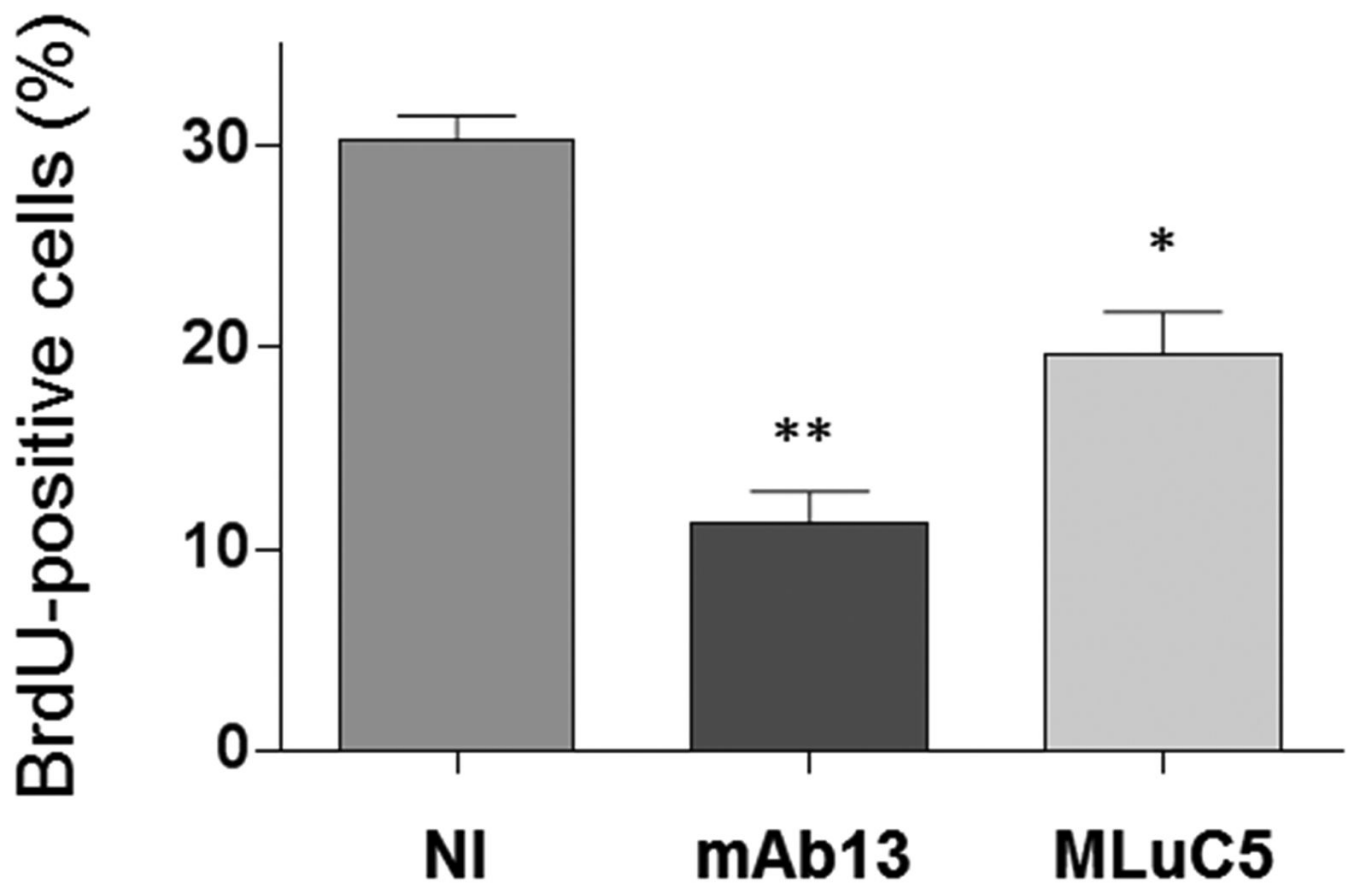
Blocking 37/67LR reduces cell proliferation. HIEC cells were treated for 1h with neutralizing-blocking anti-37/67LR antibody (MLuC5) or anti-integrin β1 antibody (mAb13) as well as non-immune serum (NI) as negative control before plating. BrdU-positive cells were counted and expressed as percentage of total cells determined by DAPI staining (mean ± SEM, n=3, * p < 0.001; ** p < 0.0001).

Taking into consideration the presence of functional membrane 37/67LR receptor for laminin adhesion in HIEC cells, we further investigated the reduction of cell proliferation in 37/67LR depleted cells observed above (Figure 6) by conducting antibody blocking experiments on wild-type HIEC cells for a 24h period followed by BrdU incorporation. As shown in Figure 8, a significant reduction of BrdU incorporation was noted with the anti-37/67LR MLuC5 azide-free antibody. The effect appears to be specific since a non-immune serum (NI) had no effect. The blocking anti-β1 integrin mAb13 antibody [71] was used as a positive control.

## Discussion

In the present study, we first analyzed the expression and function of 37/67LR in normal human intestinal epithelial cells in situ and in vitro. Previous expression studies in the human intestinal mucosa had generated conflicting results [53–55]. Our results show that the receptor is predominantly found in the epithelium in the intact intestine, being expressed in the undifferentiated and proliferative epithelial cells of both the developing and adult small intestine. This pattern of expression, which is in agreement with one of the aforementioned studies [55], confirms previous data reported in the developing and adult mouse at the transcript level [33]. Interestingly, the distribution of 37/67LR observed herein appears to be consistent with the expression of its characterized ligand LM-111, which is found in the intervillous area and the crypts before 17 weeks of gestation [48]. At later developmental stages and in the adult, LM-111 is replaced by a complementary gradient of LM-211 and LM-511 in the crypts [45,48,75]. The fact that these laminins contain the β1 chain suggests that they may serve as a ligand for 37/67LR. In this case, it would be interesting to investigate 37/67LR expression in samples from patients with Crohn’s disease since a major redistribution of these laminins has been reported in the crypts [76].

The fact that 37/67LR appears to be associated with the undifferentiated and proliferative cell population in the normal intestine is interesting in regard to the up-regulation of its expression in colorectal cancer [77–79]. To further investigate this relation, we tested HIEC cells, which are normal human intestinal crypt cells [58,60], for the expression of 37/67LR. To our knowledge, this is the first time that the expression of 37/67LR has been reported in normal human epithelial cells. Interestingly, a significant reduction of 37/67LR was noted when switching HIEC cells from a proliferative to quiescent state. This phenomenon, which has also been observed in endothelial cells [80], is consistent with the low level of 37/67LR expression observed in situ in the non-proliferative cells of the villus and lower crypt. Further analysis of its distribution revealed that a significant proportion of 37/67LR was present in the cytosol and membrane fractions. Such a distribution is consistent with previous immunolocalization studies performed on cells in culture [25] and the multiple cell functions attributed to 37/67LR in the ribosome and plasma membrane [11,12,20,21,35].

As a general strategy to investigate the role of a cell component in HIEC cells [46,47,71], we undertook the knockdown of expression of 37/67LR. A standard protocol resulted in very efficient reduction of the target gene at both the transcript and protein levels and altered the apparent viability of the cells and expression of fibronectin and IGFBP7, which are genes constitutively expressed at high levels in HIEC cells [46,47,74], confirming that the 37/67LR gene product was required for translation and general cell function [17]. Interestingly, dropping the working concentration of the siRNA targeting 37/67LR sequences by half allowed a 50% reduction of 37/67LR at the protein level without significantly altering translation efficiency as tested by the maintenance of fibronectin, IGFBP7 as well as TIMP3 expression at control levels.

A partial 37/67LR depletion protocol was then used to examine the involvement of the receptor in the regulation of cell proliferation and adhesion. A significant reduction in cell cycle progression through the G1 phase was observed as a result of partial depletion of 37/67LR in these normal intestinal cells. As proposed elsewhere [35], these data suggest that threshold levels of 37/67LR expression are required for distinct cellular functions, lower levels of 37/67LR being sufficient for normal ribosomal function but not proliferation. Taken together, these results indicate that 37/67LR regulates normal cell proliferation.

Finally, the ability of 37/67LR to interact with laminin was investigated. Laminin promotes epithelial cell adhesion in part through the CDPGYIGSR sequence on its β1 chain although its minimum ligand sequence is YIGSR [81]. Interestingly, phage-displayed mapping revealed that at least two distinct regions of 37/67LR can bind to YIGSR [82]. Partial depletion of 37/67LR in HIEC cells resulted in a significant reduction of cell adhesion to YIGSR confirming that 37/67LR is expressed in the plasma membrane under its functional form. The relatively weak adhesion to YIGSR as compared to RGD was expected considering the expression of a number of specific RGD-binding integrins in HIEC [71] and the fact that 37/67LR may act as a co-receptor with laminin-binding integrins [11], namely the α6β4 integrin [83]. By using another strategy based on the use of blocking antibodies directed toward 37/67LR, we provided evidence for the requirement of a functional form of 37/67LR at the membrane for modulating cell proliferation.

In summary, these results confirmed the predominant expression of 37/67LR in the immature intestinal epithelium and in the crypt cells of both the fetal and adult small intestine. Functionally, 37/67LR was found to be expressed in significant amounts in proliferating HIEC cells, a line of human intestinal crypt cells. Using an siRNA strategy that allowed partial down-regulation of 37/67LR expression leaving translation normal, or by using blocking antibodies, we found a significant reduction of cell proliferation and specific adhesion to the laminin-related peptide YIGSR. Taken together, these data demonstrate that 37/67LR is expressed in normal intestinal epithelial cells where it regulates cell proliferation and adhesion.
